# Neuritogenic effect of standardized extract of *Centella asiatica* ECa233 on human neuroblastoma cells

**DOI:** 10.1186/1472-6882-13-204

**Published:** 2013-08-04

**Authors:** Oraphan Wanakhachornkrai, Varisa Pongrakhananon, Preedakorn Chunhacha, Aree Wanasuntronwong, Anusara Vattanajun, Boonyong Tantisira, Pithi Chanvorachote, Mayuree H Tantisira

**Affiliations:** 1 Inter-disciplinary Program in Physiology, Graduate School, Chulalongkorn University, Bangkok 10330, Thailand; 2 Department of Pharmacology and Physiology, Faculty of Pharmaceutical Sciences, Chulalongkorn University, Bangkok 10330, Thailand; 3 Cell-based Drug and Health Product Development Research Unit, Chulalongkorn University, Bangkok 10330, Thailand; 4 Faculty of Dentistry, Mahidol University, Bangkok 10400, Thailand; 5 Department of Physiology, Phramongkutklao College of Medicine, Bangkok 10400, Thailand; 6 Faculty of Pharmacy, Silpakorn University, Nakonprathom 73000, Thailand; 7 Faculty of Pharmaceutical Scienccs, Burapha University, Chonburi 20131, Thailand

**Keywords:** Neurite outgrowth, Centella asiatica, ECa 233, ERK1/2, Akt, IMR-32 neuroblastoma

## Abstract

**Background:**

In order to gain insight into neuroprotective effects of ECa 233, a standardized extract of *Centella asiatica*, previously demonstrated in animal models of memory impairment induced by transient global ischemia or intracerebroventricular injection of β-amyloid, the effect of ECa 233 on neurite outgrowth of human IMR-32 neuroblastoma cell line was investigated.

**Methods:**

Cells were seeded and incubated with various concentrations of ECa 233. Morphometric analysis was carried out by a measurement of the longest neurite growth of cells at 24 and 48 h. Contributing signaling pathways possibly involved were subsequently elucidated by western blot analysis.

**Results:**

While ECa 233 had only limited effects on cell viability, it significantly enhanced neurite outgrowth of IMR-32 cells at the concentrations of 1–100 μg/ml*.* Western blot analysis revealed that ECa 233 significantly upregulated the level of activated ERK1/2 and Akt of the treated cells suggesting their involvement in the neuritogenic effect observed, which was subsequently verified by the finding that an addition of their respective inhibitors could reverse the effect of ECa 233 on these cells.

**Conclusions:**

The present study clearly demonstrated neurite outgrowth promoting activity of ECa 233. ERK1/2 and Akt signaling pathways seemed to account for the neurotrophic effect observed. In conjunction with *in vivo* neuroprotective effect of ECa 233 previously reported, the results obtained support further development of ECa 233 for clinical use in neuronal injury or neurodegenerative diseases.

## Background

Neurite outgrowth is an important initial step in the formation of neuronal network. Such neuronal behavior was also shown to implicate in neuronal regeneration and response to neural injury [1]. Mechanisms in regulation of neurite outgrowth have been intensively investigated and the most accepted concept involves MEK/ERK and PI3K/Akt signaling pathways. Indeed, these molecular signals are triggered by tyrosine kinase receptor activation [2-6]. Several neurotrophic ligands for tyrosine kinase receptors such as nerve growth factor (NGF), brain derived neurotrophic factor (BDNF) and neurotrophin-3 (NT-3) have been identified [7,8]. The activation of MEK/ERK and PI3K/Akt results in an increase in cell survival as well as an enhancement of neuronal growth and differentiation [9,10]. In this context, molecules or compounds that exhibit potent neurotrophic activity are of great interest and may be useful for the treatment of stroke, brain or spinal cord injury and neurodegenerative diseases [11-13].

In search for potential neurotrophic activity from herbs acting on the central nervous system, the present study focused on the neurotrophic activity of *Centella asiatica* which has, for a long time, been claimed to be beneficial for managing disorders of the brain, skin and gastrointestinal tract [14]. Additionally, it was used as a brain booster for promoting brain growth and improving memory [12]. Like many other medicinal plants, *C. asiatica* contains several active compounds, including asiatic acid, madecassic acid, asiaticoside, and madecassoside [15]. To avoid a large fluctuation in biological responses arising from variations of these bioactive constituents, we have established the standardized extract of *C. asiatica,* namely ECa 233. It is defined as a white to off-white extracted powder of *C. asiatica* containing triterpenoids not less than 80% and the ratio between madecassoside and asiaticoside was kept within 1.5 ± 0.5 [16]. Restorative and neuroprotective effects of ECa 233 have been demonstrated in animal models of learning and memory deficit induced by either a transient occlusion of common carotid arteries [17] or an intracerebroventricular injection of β-amyloid [18]. Protection of oxidative stress was proposed to be one of the possible underlying mechanisms. However, effect of ECa 233 on neurite outgrowth which could possibly be involved in its neurotrophic/neuroprotective effects has not yet been elucidated. Therefore, the present study aimed to investigate the effect of ECa 233 on the neurite growth and its underlying mechanisms in IMR-32 human neuroblastoma cells.

## Methods

### Cell culture and reagents

IMR-32 neuroblastoma cells were obtained from the American Type Culture Collection, ATCC (Manassas, VA, USA). Cells were cultured in DMEM/F12 supplemented with 10% fetal bovine serum, 2 mmol/l L-glutamine and 100 units/ml penicillin/streptomycin in a 5% CO_2_ at 37°C. BDNF, PD 098059, LY 294002 were purchased from Sigma Chemical. Inc. (St. Louis, MO, USA). Resazurin was purchased from Invitrogen (Carlsbad, CA, USA). Specific antibody for phospho-Akt, Akt, phospho-ERK1/2, ERK1/2 and GAPDH were purchased from Abcam (Cambridge, MA, USA), and peroxidase conjugated anti-rabbit secondary antibody were purchased from Cell Signaling (Danvers, MA, USA).

### Preparation of tested substances

ECa 233 containing madecassoside 52% w/w and asiaticoside 32% w/w was kindly supplied by Associate Professor Ekarin Saifah, Ph.D and collaborates, Faculty of Pharmaceutical Sciences, Chulalongkorn University. Thailand. It was suspended in sterile PBS at 10 mg/ml and served as stock solution. BDNF was dissolved in the sterile PBS to a stock solution at the concentration of 50 μg/ml. PD098059 and LY294002 were dissolved by DMSO to concentration of 0.344 and 0.267 mg/ml, respectively.

### Cell viability assay

Cells were seeded in 96-well plates and incubated for 24 h. After incubation, the plating media were removed and replaced. The cell were subsequently incubated for 30 minutes then subjected to treatments. After 24 h, cells were incubated with 1:50 resazurin at 37°C for 30 minutes. The well-plate was transferred to a fluorescence microplate reader with Softmax Pro software to measure fluorescence intensity of resorufin (resazurin product) at excitation wavelength of 530 nm and emission wavelength of 590 nm. The percentage of cell viability was calculated and compared with non-treated control. All analyses were performed for at least three independent triplicate experiments.

### Measurement of neurite outgrowth

IMR-32 cells were cultured in a 96-well culture plate. After 24 h cells were subjected to various concentrations of ECa 233 (0.1, 1, 10, 100 μg/ml) or BDNF (100 ng/ml). A neurite was identified as a process equal to or longer than cell body diameter. The cells selected randomly from 3–4 fields of each well were photographed (phase contrast, Nikon, Inverted microscope ECLIPSE Ti-u) for morphometric analyses. The length of the longest neurite from each cell was measured using NIS-Element image software [19,20].

To test the involvement of MEK and Akt pathway, their respective inhibitors, PD098059 (5 μM) for MEK and LY294002 (7.5 μM) for PI3K/Akt, was added at 30 min prior to the test substance.

### Western blot analysis

After specified treatments, cells were incubated in lysis buffer containing 20 mM Tris–HCl (pH 7.5), 1% TritonX-100, 150 mM sodium chloride, 10% glycerol, 1 mM sodium orthovanadate, 50 mM sodium fluoride, 100 nM phenylmethylsulfonyl fluoride, and a commercial protease inhibitor cocktail (Roche Molecular Biochemicals, Indianapolis, IN, USA) for 30 minutes on ice. Cell lysates were collected and centrifuged 12,000 rpm at 4°C, the supernatant was collected and the protein content was determined using Bradford method (Bio-Rad, Hercules, CA, USA). Equal amount of proteins from each sample were denatured by heating at 95°C with laemmli loading buffer for 5 min and were subsequently loaded onto 10% SDS-PAGE. After separation, proteins were transferred onto 0.45 μm nitrocellulose membranes (Bio-Rad). The transferred membranes were blocked in 5% non-fat dry milk in TBST (25 mM Tris–HCl (pH 7.5), 125 mM NaCl, 0.1% tween20) for 1 h. Then washed with TBST and further incubated with the indicated primary antibodies at 4°C overnight. Membrane were washed twice with TBST for 10 min and incubated with secondary antibody for 1 h at room temperature. The immune complexes were detected by enhanced chemiluminescence substrate (Supersignal West Pico; Pierce, Rockford, IL, USA) and quantified using Image J software.

### Data analysis

The results are expressed as mean ± S.D. or S.E. Simple comparison between two groups was performed using *t*-test. Comparison of data between groups was performed using one-way ANOVA follow by Tukey’s post hoc test. *P* < 0.05 was considered statistically significant.

## Results

### Effect of ECa 233 on viability of IMR-32 cells

**Figure 1:**
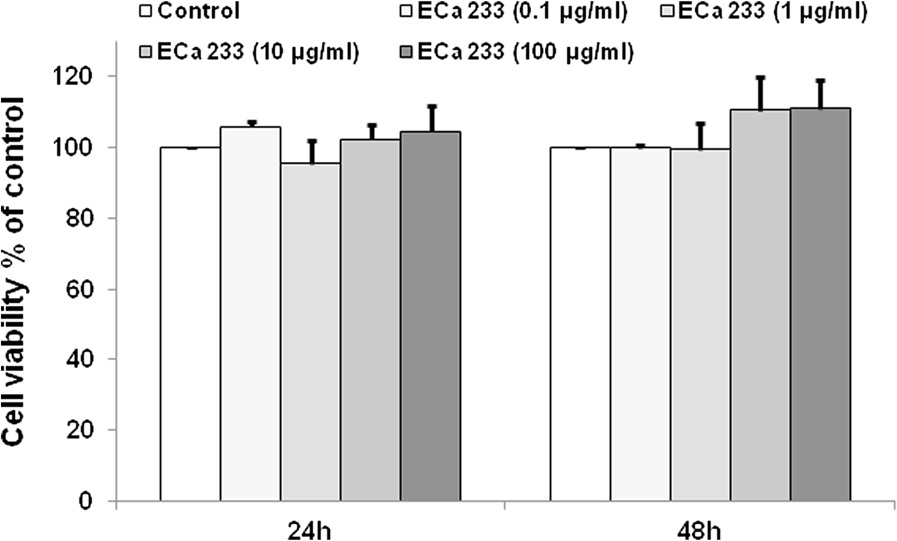
**Effects of ECa 233 on cell viability and proliferation of IMR-32 cells at 24 and 48 h after applying.** There was no significant difference among groups.

First, effect of ECa 233 on cell viability of IMR-32 cells was determined using Resazurin based assay. Cells were incubated in the presence or absence of ECa 233 (0.1, 1, 10 and 100 μg/ml) for 24 and 48 h and percentage of cell viability was determined. Percentage of such cell viability was compared among groups as well as with non-treated control (p < 0.05) (Figure 1). The results show that cell viability was 106 ± 1.6, 95.6 ± 6.44, 102.4 ± 4.03 and 104.6 ± 7.48% of control in response to ECa 233 at the concentrations of 0.1, 1, 10 and 100 μg/ml, respectively. In addition, cell viability at 48 h after treatment in all ECa 233-treated groups showed no significant change from that of non-treated control indicating that treatment with ECa 233 at the concentrations tested had neither cytotoxic nor proliferative effects on these neuroblastoma cells.

### Neuritogenic activity of ECa 233 on neurite outgrowth

**Figure 2:**
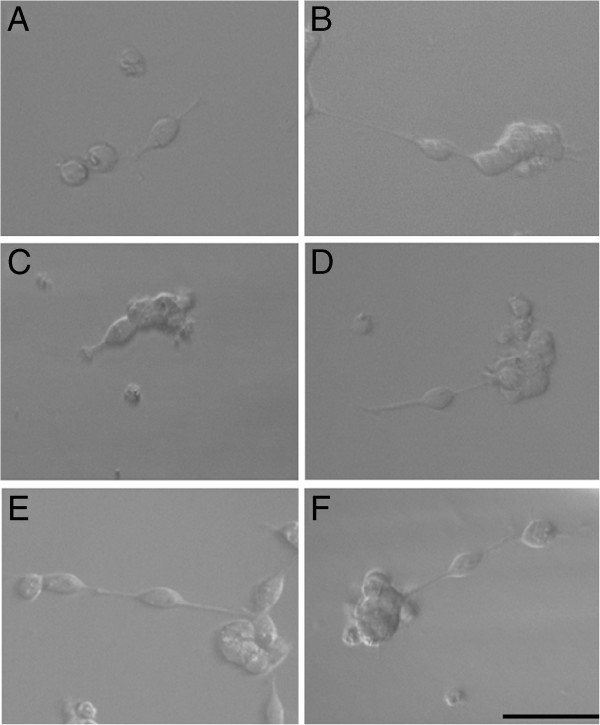
**Representative photomicrograph of IMR-32 cells using phase contrast. (A)** non-treated control, **(B)** BDNF 100 ng/ml, **(C-F)** ECa 233 0.1, 1, 10 and 100 μg/ml respectively (scale bar = 50 μm). Cells treated with BDNF and ECa 233 at the concentrations of 1, 10 and 100 μg/ml showed relatively longer neurite length.

The non-cytotoxic and non-proliferative concentrations of ECa 233 at 0.1, 1, 10 and 100 μg/ml were further used in neurite outgrowth experiments. BDNF, a well-known trophic factor of neurite extension and neuronal survival was used as a positive control. We found that the addition of 100 ng/ml BDNF to the cells caused dramatic increase of the neurite outgrowth (Figure 2B). Likewise, cells treated with ECa 233 at the concentrations of 1, 10 and 100 μg/ml exhibited increased length of neurite compared to those of non-treated control (Figure 2D-F).

**Figure 3:**
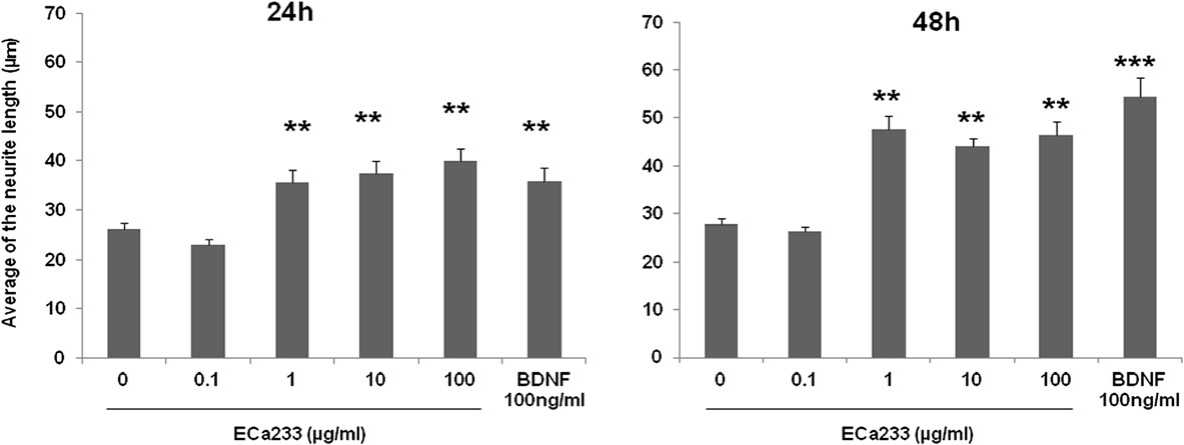
**Morphometric analysis of IMR**-**32 cells affected by ECa 233.** After cells cultured for 24 and 48 h in the presence of either ECa 233 (0.1, 1, 10 and 100 μg/ml) or BDNF (100 ng/ml). Morphometric analysis was carried out by measuring neurites from 60 living cells per treated condition, (n = 3). Average of the neurite length was significantly increased after being treated with ECa 233 (1, 10 and 100 μg/ml) or BDNF. The data presented as mean ± S.E. ** = *p* < 0.01, *** = *p* < 0.001 vs non-treated control.

The morphometric analysis was carried out by measuring the longest neurite length per cell (Figure 3). After 24 and 48 h of treatment, the length of neurite obtain from cells treated with ECa 233 at the concentration of 0.1 μg/ml (23.02 ± 1.05 and 26.46 ± 0.87 μm) was not significantly different from those of non-treated cells (26.22 ± 1.19, 27.91 ± 1.01 μm). Whereas, neurite lengths exposed to ECa 233 at the concentrations of 1, 10 and 100 μg/ml were significantly enhanced in a time-dependent manner (35.58 ± 2.61, 37.39 ± 2.69 and 39.9 ± 2.73 μm at 24 h, and 47.57 ± 2.84, 44.03 ± 1.66 and 46.52 ± 2.71 μm at 48 h). Similar results were obtained when the cell were treated with BDNF 100 ng/ml (35.83 ± 2.17 and 54.38 ± 4.06 μm). Notably, the effect of ECa 233 at 1, 10 and 100 μg/ml was comparable to that of BDNF with no significant difference.

### Possible mechanisms of ECa 233-mediated neuritogenic activity

#### Effect of ECa 23 on ERK1/2 and Akt

**Figure 4:**
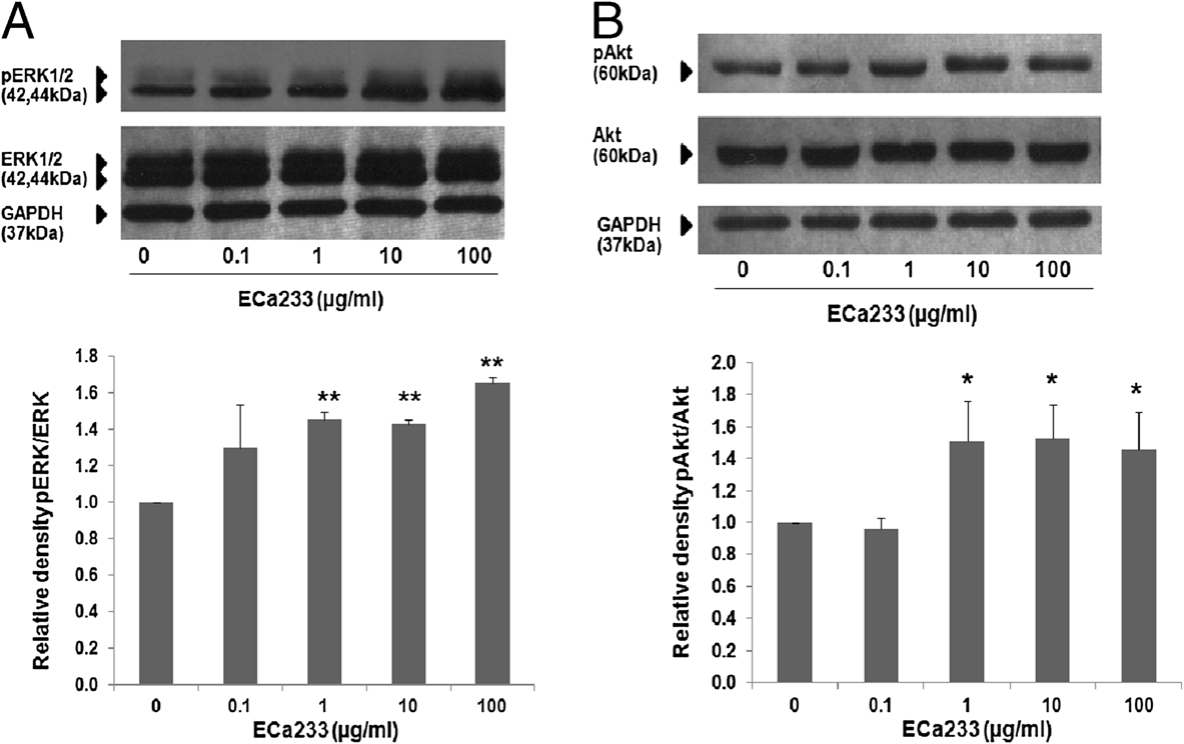
**Relative blot density of pERK/ERK and pAkt/Akt in response to ECa 233 treatment.(A)** ERK1/2 and ERK1/2 phosphorylation **(B)** Akt and Akt phosphorylation. The level of phospho-ERK1/2 and phospho-Akt in IMR-32 cells after treated with ECa 233 (1, 10 and 100 μg/ml) for 6 h were significantly higher than that of control. Densitometric analysis of the indicated proteins normalized with the density of GAPDH band were plated and presented as mean ± S.D. of three independent experiments, * = *p* < 0.05, ** = *p* < 0.001 vs non-treated control.

In order to provide the possible mechanism of ECa 233-mediated neurite outgrowth observed in IMR-32 cells, western blot analysis was carried out to determine whether ERK and Akt signaling were involved. Cells were treated with ECa 233 and the level of phosphorylated ERK and phosphorylated Akt were determined. Figures 4A and B demonstrated that relative blot density of pERK/ERK and pAkt/Akt in response to ECa 233 at the concentration of 0.1 μg/ml was not different from that of non-treated group. However, ECa 233 at the concentrations of 1, 10 and 100 μg/ml significantly increased the level of phosphorylated ERK and phosphorylated Akt, suggesting that ECa 233 could possibly increase the neurite outgrowth via these 2 major pathways.

#### Effect of ECa 233 on neurite outgrowth in the presence of PD098059 and LY294002

**Figure 5:**
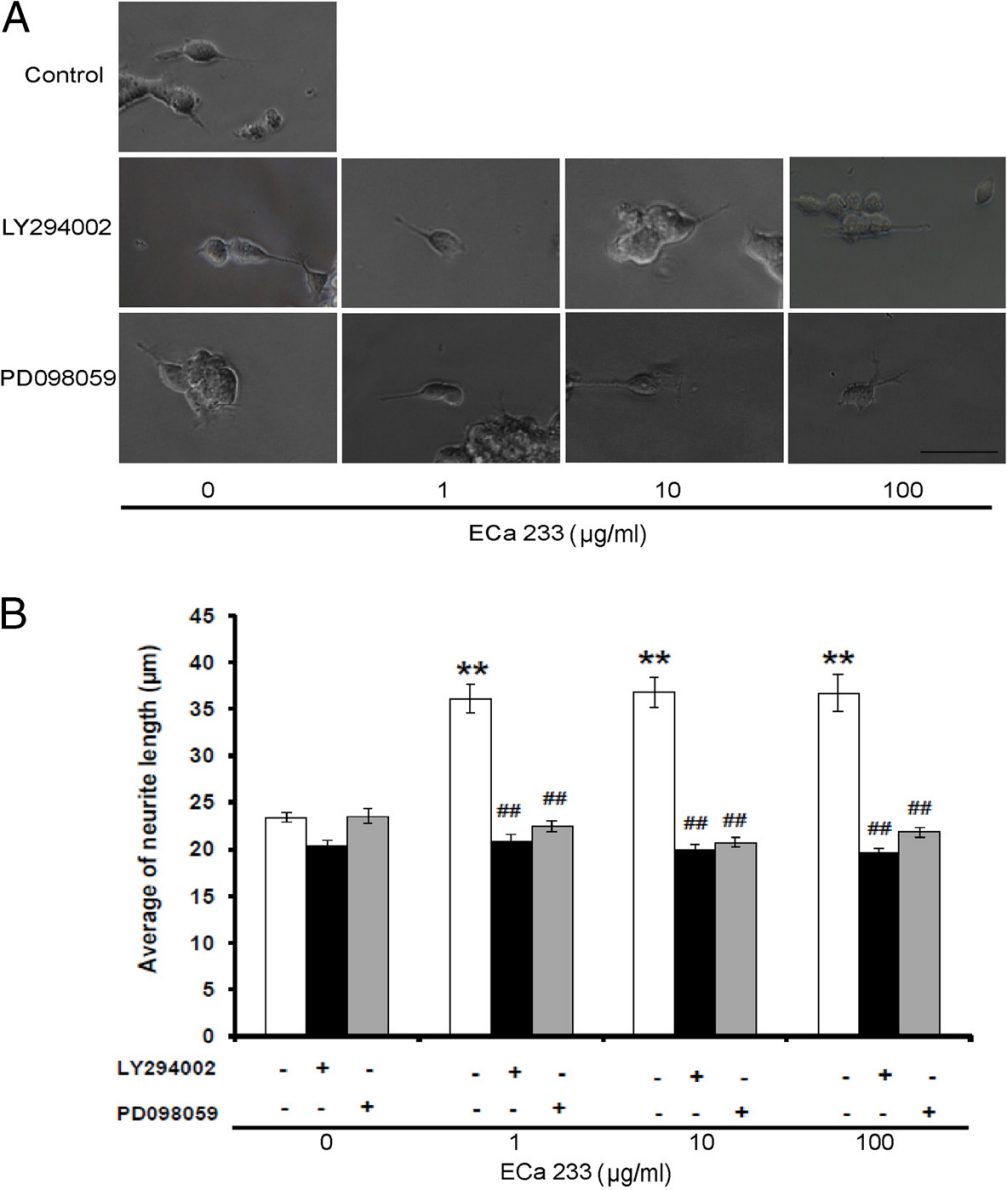
**Representative photomicrograph of IMR**-**32 cells.(A)** after pre-treated with LY294002 or PD098059 in the presence or absence of ECa 233 (1, 10 and 100 μg/ml) (scale bar = 50 μm). (**B**) Morphometric analysis of the cell treated with LY294002 or PD098059 on the increment of neurite outgrowth induced by ECa 233. Neurites from 60 living cells per treated condition were evaluated, (n = 3). Data are presented as mean ± S.E., ** = *p* < 0.001 vs non-treated control. ^##^ = *p* < 0.001 vs ECa 233 treated groups.

Subsequently, the involvement of ERK and Akt signaling pathways in the neurite outgrowth promoting activity of ECa 233 was confirmed by treatment of specific inhibitor of MEK (PD098059) or PI3K (LY294002) prior to the exposure to ECa 233. In comparison to neurite lengths of non-treated group which was 23.39 ± 0.54 μm (Figure 5A), ECa 233 at the concentrations of 1, 10 and 100 μg/ml significantly enhanced the neurite outgrowth into 36.12 ± 1.53, 36.79 ± 1.60 and 36.69 ± 1.98 μm respectively. Pretreatment of the cell with PD098059 significantly decreased the neurite outgrowth of the respective concentrations of ECa 233 into 22.45 ± 0.65, 20.76 ± 0.532 and 21.83 ± 0.53 μm. Similar results were demonstrated by LY294002 which significantly decreased the ECa 233-induced neurite outgrowth into 20.83 ± 0.81, 20.03 ± 0.49 and 20.76 ± 0.53 μm, respectively (Figure 5B). As expected, PD098059 (5 μM) or LY294002 (7.5 μM) in the concentration that exhibited no significant effect on neurite outgrowth (23.52 ± 0.84 and 20.43 ± 0.54 μm, respectively) completely abolished neurite stimulating effect of ECa 233.

## Discussion

Although the plant originated extracts have garnered attentions as important sources of drugs and health products, the inconsistency of main components found in such extracts limits their use as well as further development to clinical use. Establishment of standardized extract with consistency of its bioactive constituents could enhance reproducible biological responses and subsequently reliable use of herbal products [21]. Based on well-defined constituents and numbers of scientific evidence, EGb 761, a standardized extract of Chinese ancient plant, *Ginko biloba*, known as a brain tonic remedy, has been prescribed for memory deficit all over the world [22]. By the same analogy, *C. asiatica* which has long been used in Ayurveda to enhance memory was extensively investigated [23]. In an attempt to make a reliable herbal product, ECa 233, a standardized extract of *C. asiatica*, was established and investigated for its effect on brain function. We have found that ECa 233 could attenuate memory deficit and exerted anxiolytic effect in animal models [16-18]. As administration of fresh leaf juice of *C. asiatica* was found to enhance dendritic aborization of hippocampal and amygdala neurons in neonatal and adult rats [24-27], we hypothesized that ECa 233 might enhance neurite outgrowth.

As expected, the present study clearly demonstrated neuritogenic effect of ECa 233 in the range of 1–100 μg/ml. Further experiment was then conducted to gain an insight into signaling pathways possibly involved. Numerous signaling pathways involving in neurite outgrowth have been proposed [2,5]. Among them, ERK and Akt activations have long been known to be important for neurite elongation induced by certain neurotrophic factors including NGF [28,29], neurotrophin-3 (NT-3) [30], BDNF [31] as well as natural compounds including FK506 [32,33], genipin [34], honokiol [4] and militarinone A [9]. In addition, ginsenoside, triterpenoidglycoside from gingseng, has been shown to significantly increase the neurite outgrowth of neuroblastoma cells [35].

In line with the findings previously demonstrated by some other natural products mentioned above, western blot analysis demonstrated that administration of ECa 233 significantly increased the level of phosphorylated ERK and Akt in IMR-32 cells indicating their activation in the presence of ECa 233. In addition, contribution of ERK and Akt signaling pathways was subsequently confirmed by the results that the neurite outgrowth promoting effect of ECa 233 was abolished by specific inhibitors of MEK (PD098059) or PI3K (LY294002) suggesting that ECa 233 promoted neurite outgrowth in human neuroblastoma IMR-32 cells via MEK/ERK and PI3K/Akt-dependent mechanisms. Though, asiatic acid, one of chemical constituents present in *C. asiatica* has been proposed to elicit its effect via MAP kinase pathway [12], increases of phosphorylated ERK and Akt observed in the present study are likely to be attributable to madecassoside and/or asiaticoside, the major constituents of ECa 233.

In agreement with the fact that *C. asiatica* is widely consumed as food, ECa 233 in the concentrations that promoted neurite outgrowth in human neuroblastoma IMR-32 cells had no effect on cell viability under the experimental condition indicating safety of ECa 233, a standardized extract derived from *C. asiatica*. Similarly, safety profiles of ECa 233 have been previously reported in both acute and sub-chronic toxicity testing. Oral administration of ECa 233 in the dose up to 10 g/kg did not cause any lethality and no significant adverse effects was observed in experimental animals receiving daily administration of ECa 233 in the dose range of 10–1000 mg/kg/day [36]. In consideration to very favorable safety profiles and significant *in vivo* neuroprotective activity of ECa 233 [18], the results of the present study supports further development of ECa 233 for neuronal injury as well as neurodegenerative diseases in human.

## Conclusions

The present study demonstrated the neurite outgrowth promoting activity of ECa 233 in human IMR-32 neuroblastoma cell line. The neuritogenic effect observed seemed to be mediated via ERK1/2 and Akt signaling pathways. The results obtained support potential benefit of ECa 233 for the management of neuronal injury and neurodegenerative diseases. Further pharmacodynamic and pharmacokinetic studies of ECa 233 are needed to bring ECa 233 into clinical use.

## Abbreviations

C. asiatica: Centella asiatica; MEK: Mitogen activated protein kinase; ERK: Extracellular signaling regulated kinase; PI3K: Phosphoinositide-3-kinase; NGF: Nerve growth factor; BDNF: Brain derived neurotrophic factor; NT-3: Neurotrophin-3; PD: PD 098059; LY: LY 294002.

## Competing interests

All authors declare that they have no competing interests.

## Authors’ contributions

OW participated in design and conducting the experiments, analysis of data, drafting the manuscript. VP, PrC and AW participated in technical supports and interpretation of the data. AV and BT supervised, design of experiments, analyzed and interpretation of the data. PiC was a coordinator, designed the study, analysis and interpretation of data, and drafting the manuscript. MT supervised, conceived and designed the experiment, interpretation of the data and revision of the manuscript. All authors have read and approved the final manuscript.

## Pre-publication history

The pre-publication history for this paper can be accessed here:

http://www.biomedcentral.com/1472-6882/13/204/prepub
